# Involvement of miR-126 in autoimmune disorders

**DOI:** 10.1186/s12948-018-0089-4

**Published:** 2018-05-02

**Authors:** Marco Casciaro, Eleonora Di Salvo, Teresa Brizzi, Carmelo Rodolico, Sebastiano Gangemi

**Affiliations:** 1 School and Division of Allergy and Clinical Immunology, Department of Clinical and Experimental Medicine, Messina University Hospital, 98125 Messina, Italy; 2 Department of Chemical, Biological, Pharmaceutical and Environmental Sciences, University of Messina, Messina, Italy; 3 Department of Clinical and Experimental Medicine, University of Messina, 98125 Messina, Italy; 4 Operative Unit of Allergy and Clinical Immunology, Department of Clinical and Experimental Medicine, University of Messina, Messina, Italy

## Abstract

**Background:**

Micro-RNA represent a great family of small non-condign ribonucleic acid molecules; in particular microRNA-126 is an important member of this family and is expressed in many human cells such as cardiomyocytes, endothelial and lung cells. Some studies have shown the implication of miR-126 in cancer, but recently significant progresses have also been made in determining the role of miR-126 regulating immune-related diseases; probably, in a near future, they could potentially serve as diagnostic biomarkers or therapeutic targets.

**Objective:**

The purpose of this review is to investigate the role of miR-126 in autoimmune diseases, so as to offer innovative therapies.

**Results:**

According literature, it was concluded that miRNAs, especially miR-126, are involved in many pathologies and that their expression levels increase in autoimmune diseases because they interfere with the transcription of the proteins involved. Since microRNAs can be detected from several biological sources, they may be attractive as potential biomarkers for the diagnosis, prognosis, disease activity and severity of various diseases. In fact, once confirmed the involvement of miR-126 in autoimmune diseases, it was speculated that it could be used as a promising biomarker. These discovers implicate that miR-126 have a central role in many pathways leading to the development and sustain of autoimmune diseases. Its key role make this microRNA a potential therapeutic target in autoimmunity.

**Conclusion:**

Although miR-126 relevant role in several immune-related diseases, further studies are needed to clear its molecular mechanisms; the final step of these novel researches could be the blockage or the prevention of the diseases onset by creating of new targeted therapy.

## Background

Micro-RNA represent a great family of small non-condign ribonucleic acid molecules (RNA) [1]; in particular microRNA-126 (miRNA-126, miR-126) is an important member of this family, encoded by 7th intron of the EGFL7 gene in human chromosome 9q34.3 and expressed in many humans cells such as cardiomyocytes, endothelial and lung cells [2].

miR-126 aroused many interest, especially in the epigenetic studies, because of its ability to bind directly the DNA, preventing the transcription, translation and degradation of mRNA [3]. As described above, miR-126 and EGFL7 are intimately correlated [2]; EGFL7 results to be involved in the balance of blood vessels, cell migration and maturation/formation of T-helper 2 cells. These conditions make EGFL7 and miR-126 targets of relevant importance for many diseases such as tumours and autoimmune diseases [4].

Some studies shown the implication of miR-126 in cancer; recently, significant progress have also been made in determining the role of miR-126 in the regulation of the immune-related diseases [5–7]. As reported by some researchers, microRNAs are involved in the immune response and are associated to autoimmune diseases (i.e. Lupus, Rheumatoid Arthritis) [5]; probably, in a near future, they could potentially serve as diagnostic biomarkers or therapeutic targets [2].

The purpose of this review is to investigate the role of miR-126 in autoimmune diseases so as to offer innovative therapies.

## Autoimmune diseases related to miR-126

miR-126 are implicated in inflammatory and angiogenetic processes. Through these processes, miR-126 plays a role in cancer and autoimmune biology. The microRNA 126 was shown to have important roles in cancers of the gastrointestinal and genital tracts, in the cancers of the breast, the thyroid, the lung and in some other ones [8–14]. Usually, miR-126 is down-regulated in tumours, most likely due to its ability to inhibit malignant cell growth, adhesion, migration, and invasion through suppressing a range of important target genes. Also, reduced levels of miR-126 are a significant predictor of poor survival in neoplastic patients [15]. Furthermore, microRNA-126 expression was associated to autoimmune diseases (Table 1) suggesting that this miRNA could represent a common therapeutic target in the above cited disorders.

**Table 1:** miR-126 related autoimmune diseases

miR-126 related autoimmune diseases
Systemic lupus erythematosus
Rheumatoid arthritis
Dermatomyocytes
Multiple sclerosis
Psoriasis
Cardiomyopathies

### Rheumatoid arthritis (RA)

Rheumatoid arthritis is an autoimmune disease that cause non-septic proliferative synovitis and may compromise the integrity of bone and cartilage tissues, thus causing articular dysfunction [16]. Epigenetics (chromatin rearrangement, histone modification and DNA methylation) have an important role in the occurrence and development of various autoimmune diseases [17–21]. Genomic hypomethylation of T cells in RA patients can provoke the overexpression of some interleukins; in example, the interleukin-8 (IL-8) is overproduced by CD4+ T cells after the hypomethylation of its gene promoter [22]. Moreover, the methylation of FOXP3 gene promoter region in CD4+ and CD25+ T cells is correlated to RA [23]. Other DNA methylation sensitive genes were also identified (CD11a, CD70, CD10L/IgEFcRYI and perforin) [24–27]. Yang et al. results showed elevated mRNA and protein expression levels of both CD11a and CD70 in RA patients derived CD4+ T cells tranfected with miR-126 plasmids, along with depressed DNMT1 protein but not mRNA levels. The introduction of a miR-126 inhibitor reversed such effects, suggesting the potential of elevated miR-126 expression in inducing the hypomethylation of CD11a and CD70 genes. Probably the depression of DNMT1 protein could cause the over-expression of CD11a and CD70, leading to the onset and progression of RA [28]. Tumour necrosis factor alpha (TNFα), a key player in the development of RA, is known to induce macrophages and other immune system cells to produce pro-inflammatory mediators (IL-1, IL-6 and IL-8) [29]; it also leads to T cell activation, and stimulate endothelial cells to express adhesion molecules [29]. According Castro et al. the anti-TNFα therapy in RA patients upregulated their serum miR-126 profile; moreover, they found that miR-126 correlated to changes in the inflammatory parameters (CRP or ESR) [30]. Another main pathophysiological aspect of RA is the high increase of resident synovial cells, also known as synovial fibroblasts (SFs, RASFs). During the inflammation process, SFs become hyperplastic, invasive, and highly migratory, reminiscent of tumour cells, and have a fundamental role in the pathogenesis of RA [31–33]. Phosphoinositide 3-kinase/protein kinase B (PI3K/AKT) signalling pathway was reported in SFs; there, it showed an unusual activation state, which might lead to the imbalance of SFs proliferation and apoptosis. It was also shown that the gene coding for the PIK3R2 was targeted by microRNA-126 [34, 35]. Gao et al. aimed to explore the associations between miR-126, PIK3R2 gene and PI3K/AKT signalling pathway in RASFs [36]. In fact, direct targeting of SFs in RA was proposed as an objective for new therapies. Gao et al. showed that miR-126 targeting PIK3R2 could stimulate growth and apoptosis resistance of SFs by regulating PI3 K/AKT signalling pathway in RA [36]. In addition, RASFs can also trigger the production of innate immunity products [37]. Qu et al. aimed to deepen the influence of miR-126 on the cellular cycle of RASFs. As they reported, miR-126 regulated the PI3K-AKT signalling pathway, through improving proliferation and blocking apoptosis. A higher miR-126 expression could augment growth and proliferation of RASFs by inhibiting cell apoptosis and functions influencing the cycle from G0/G1 phase to S phase [38]. High serum levels of miR-126-3p in RA patients were also confirmed by Murata et al. [39]. Cysteine-rich 61 (CCN1) is an important pro-inflammatory cytokine in RA, so Cheng et al. studied its role in the angiogenesis, a detrimental event in the disease. According their results, CCN1 markedly repressed miR-126 expression in osteoblasts. Co-transfection of cells with miR-126 mimic abolished CCN1-induced VEGF production and angiogenesis [40].

### Systemic lupus erythematosus (SLE)

SLE is a severe autoimmune disease caused by many factors such as innate and adaptive immune system dysfunction, genetic predisposition, external environmental factors and hormonal dysfunctions [41, 42]. The researches by Zhao’s group on CD4+ demonstrated that miRNAs play a central role in SLE. miR-126 and miR-148a, directly inhibited the enzyme DNA methyltranferase 1 (DNMT1) binding to the 3′UTR of the transcript. This block caused the over-expression of miR-126 and then an over-production of CD4+ cells that stimulated IgG production with a consequent worsening of the disease [2, 43]. Different results were obtained from a study on serum; Liu et al. confirmed that the inappropriate activation of interferon-alpha (IFN-⍺) caused the onset of the disease. In fact, their study showed that plasma levels of IFN-alpha in SLE patients were significantly higher than in controls, suggesting that the IFN pathway is closely associated to SLE. In SLE patients’ serum, low levels of miR-126 were also reported. Thus, in this study, the correlation between miR-126 and SLE was clarified by using a miR-126 inhibitor; it was observed that the start and the development of the disease, while miR-126 level was lower, were worse; they speculated that miR-126 can suppress the development of SLE inhibiting IFN pathway signalling [44].

### Multiple sclerosis (MS)

Multiple sclerosis is a chronic inflammatory disease with an autoimmune basis leading to the demyelination of the central nervous system [45, 46].

A close correlation between this pathology and miR-126 was observed by Chen et al. because of the higher levels of the microRNA in MS patients [47]. Another study showed that miR-126 significantly reduced the adhesion of leukocytes to the brain lesions involved in the disease by directly controlling some molecules fundamental for the migration of leukocytes into the central nervous system (CNS) [48]. About the molecules involved, Ets1, controlled by TNF-alpha, resulted to influence the cytokines and the chemokines essential for neuroinflammation by intervening on the EGFL7; less directly, the diminution of the expression of miR-126 could also be controlled by other microRNAs (miR-155 and miR-146a) acting on VCAM1 which in turn acts on the transcription factor Ets1; also the expression of Nf-kB controls the regulation of miR-146a and thus indirectly could control the expression of miR-126 [48].

### Psoriasis

Psoriasis is a chronic inflammatory skin disease caused by an immune system alteration and that occurs with severe skin lesions. Many studies have highlighted the involvement of over 1000 genes, described in psoriasis lesions [49–51]. García-Rodríguez et al. demonstrated the involvement of miRNAs in controlling the gene expression of inflammatory proteins in psoriatic skin. In particular, their research showed that levels of miR-126 in serum of patients with chronic inflammatory diseases were very high but without a close correlation between miR-126 and psoriasis. Nevertheless, miR-126 analysis was useful to clarify the role that miRNAs play as gene regulatory molecules [49].

Pivarcsi et al. knowing that the levels of circulating miRNAs were altered in psoriasis, suggested to use a new biological therapy (anti-TNF-alpha) in order to decrease miRNAs serum levels (including miR-126) in psoriatic patients. They reported the correlation between TNF-alpha and miR-126, together with its importance in regulating immune system cells, and the possible use of miRNAs as biomarkers [52].

### Cardiomyopathies

Cardiomyopathies are a group of diseases influenced by the intervention of immune system cells leading to an impaired function of the myocardium. miRNAs were found being abundant in biological fluids, especially in various pathological conditions such as in cardiovascular disease [53–55].

Satoh et al. demonstrated that Toll-like receptor (TLR) 4 is implicated in the aetiology of human dilated cardiomyopathy (DCM) and that miR-126 and other miRNAs directly regulate TLR4 expression. In fact, they speculated a pathophysiological correlation between high levels of miR-126 and cardiomyopathy immune response [53].

### Dermatomyositis

Kim et al. studied miR-126 and its role in juvenile dermatomyositis (DM). In DM, miR-126 was found to be down-regulated, explaining the perimysial and endomysial damages implicated in the processes of inflammation and angiogenesis [56]. Probably, through these interventions, miR-126 is also implicated in the development of part of the autoimmune diseases mentioned in the above paragraphs.

## Discussion

Based on the data obtained from literature results, it was concluded that microRNAs are involved in many disorders [57–60]. Their expression levels increase in autoimmune diseases because they interfere with the transcription of the proteins involved [61].

**Fig. 1:**
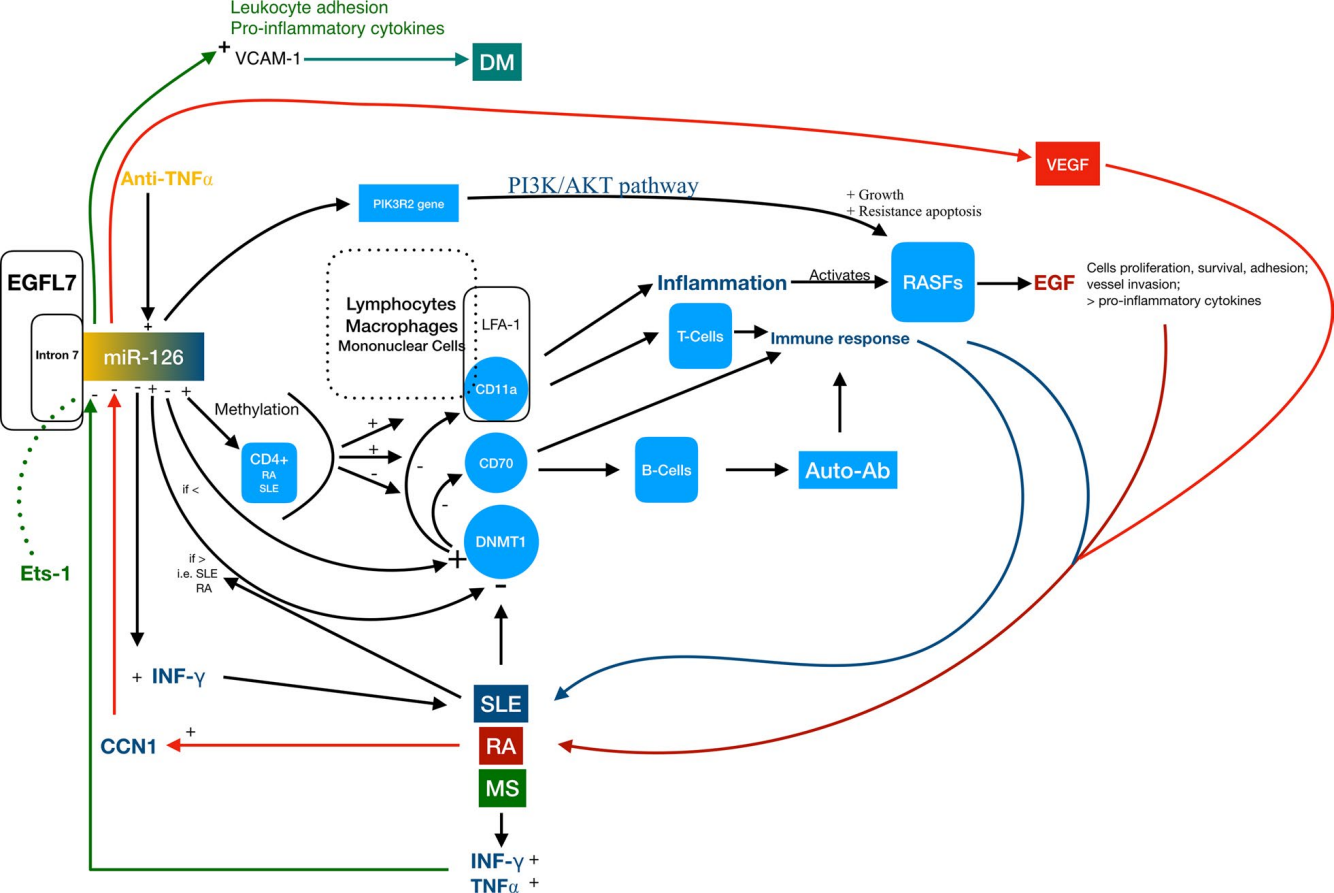
miR-126 physio-pathological role in immune disorders

Yang et al. determined miR-126 involvement in inducing hypomethylation of CD11a and CD70 genes, possibly via depressing DNMT1 protein levels, thus causing over-expression of CD11a and CD70. These CDs are largely known to enhance inflammatory response by influencing T cells activity [2, 27, 28, 43]. However, the consequent over expression of cytokines could also promote B cells activity and the production of autoantibodies with the occurrence of auto-immune diseases. Furthermore, CD70 as a co-stimulator of B cells, if augmented, could induce the production of autoantibodies itself [62]. miR-126 down regulation confirmed its central role in RA due to its capacity to inhibit PI3K/AKT signalling pathway and disrupting the balance of RASFs cellular cycle by targeting *PIK3R2* [36]. MicroRNA 126 confirmed its importance in the development of these inflammatory diseases; in fact, high miR-126 levels abolished CCN1-induced VEGF production and angiogenesis [40]. On the contrary, serum levels of the above cited microRNA did not match with those present in cells or tissue of autoimmune patients; in particular, low levels of miRNA-126 corresponded to high levels of IFN-γ suggesting another important physio-pathological target [44]. Another important discover was that high TNFα and IFNγ levels downregulated the concentration of brain endothelial miR-126. TNF-⍺ could induce ets-1 and in turn modulate Egfl7 and consequently miR-126 [48] (Fig. 1).

According data collected, since microRNAs can be detected from several biological sources, they may be attractive as potential biomarkers for the diagnosis, prognosis, disease activity and severity in several diseases [63]. In fact, once confirmed the involvement of miR-126 in autoimmune diseases, it was clear that it could also be used as a promising biomarker.

Previous studies also showed how miR-126 expression could be influenced by modern therapies. Meira et al. analyzed MS patients and confirmed the up-regulation of miR-126 in the immune disease. In addition, they showed that natalizumab (a inhibitory molecule that binds α4β1- and α4β7 integrins) could influence miR-126 expression, normalising it [64]. In particular, Meira and colleagues evaluated the expression of miR-126 in CD4+ T cells obtained from the serum of natalizumab-treated patients noticing a significant down-regulation of the miRNA [64]. Another group demonstrated the important role of Sifalimumab (a human anti–IFN monoclonal antibody which specifically neutralizes most IFN subtypes, preventing signaling through the type I IFN receptor) in SLE. They showed that Sifalimumab decreased miR-126 expression in SLE [65].

## Conclusion

These discovers implicate that miR-126 have a central role in many pathways leading to the development and the sustain of autoimmune diseases. Its key role could let us speculate this microRNA to be a potential therapeutic target in autoimmunity. All the papers reviewed emphasize its relationship with the innate and the adaptive immune response. More pathophysiological studies should consider it as the fulcrum for possible therapeutic approaches. Although miR-126 relevant role, further researches could be useful to find many of its molecular mechanisms in order to block or prevent the onset of the diseases by considering targeted therapy; biological drugs/monoclonal therapies together with the transfection with miRNA plasmids or the usage of inhibitors could represent a path to follow. Decreasing the side effects while obtaining more effective treatments for patients could be an achievable objective.
